# Risk of dietary and breastmilk exposure to mycotoxins among lactating women and infants 2–4 months in northern India

**DOI:** 10.1111/mcn.13100

**Published:** 2020-11-16

**Authors:** Rukshan V. Mehta, Anthony J. Wenndt, Amy Webb Girard, Sunita Taneja, Samriddhi Ranjan, Usha Ramakrishnan, Reynaldo Martorell, P. Barry Ryan, Kannan Rangiah, Melissa F. Young

**Affiliations:** ^1^ Doctoral Program in Nutrition and Health Sciences, Laney Graduate School Emory University Atlanta Georgia USA; ^2^ The Hubert Department of Global Health, Rollins School of Public Health Emory University Atlanta Georgia USA; ^3^ School of Integrative Plant Science & Tata Cornell Institute Cornell University Ithaca New York USA; ^4^ Centre for Health Research and Development Society for Applied Studies New Delhi India; ^5^ Department of Environmental Health, Rollins School of Public Health Emory University Atlanta Georgia USA; ^6^ Council for Scientific and Industrial Research Central Food Technological Research Institute Mysuru India

**Keywords:** aflatoxins, breast milk, cereal crops, deoxynivalenol, fumonisins, ochratoxins

## Abstract

Mycotoxins are carcinogenic secondary metabolites of fungi that have been linked to infant growth faltering. In this study, we quantified co‐occurring mycotoxins in breast milk and food samples from Haryana, India, and characterized determinants of exposure. Deterministic risk assessment was conducted for mothers and infants. We examined levels of eight mycotoxins (Aflatoxin B_1_, B_2_, G_1_, G_2_, M_1_, M_2_; Ochratoxin A, B) in 100 breast milk samples (infants 2–4 months) using ultra‐high‐performance liquid chromatography tandem mass spectrometry. Aflatoxin B_1_ (AFB_1_), fumonisin B_1_ (FB_1_) and deoxynivalenol (DON) were detected in several food items (*n* = 298) using enzyme‐linked immunosorbent assays. We report novel data on the presence of mycotoxins in breast milk samples from India. Whereas breast milk concentrations (AFM_1_ median: 13.7; range: 3.9–1200 ng/L) remain low, AFM_1_ was detected above regulatory limits in 27% of animal milk samples. Additionally, 41% of infants were above provisional maximum tolerable daily intake (PMTDI) limits for AFM_1_ due to consumption of breast milk (mean: 3.04, range: 0.26–80.7 ng kg^−1^ bw day^−1^). Maternal consumption of breads (*p* < 0.05) was associated with breast milk AFM_1_ exposure. AFB_1_ (μg/kg) was detected in dried red chilies (15.7; 0–302.3), flour (3.13; 0–214.9), groundnuts (0; 0–249.1), maize (56.0; 0–836.7), pearl millet (1.85; 0–160.2), rice (0; 0–195.6), wheat (1.9; 0–196.0) and sorghum (0; 0–63.5). FB_1_ (mg/kg) was detected in maize (0; 0–61.4), pearl millet (0; 0–35.4) and sorghum (0.95; 0–33.2). DON was not detected in food samples. Mothers in our study exceeded PMTDI recommendations for AFB_1_ due to consumption of rice and flour (mean: 75.81; range: 35.2–318.2 ng kg^−1^ bw day^−1^). Our findings show the presence of Aflatoxin B_1_ and M_1_ at various levels of the food chain and in breast milk, with estimated intakes exceeding PMTDI recommendations. Aflatoxins are known carcinogens and have also been linked to stunting in children. Their presence across the food system and in breast milk is concerning, thus warranting further research to replicate and expand on our findings and to understand implications for maternal and child health.

**Key Messages**

- AFM_1_ was detected in 41% of breast milk samples (median: 13.7, range: 3.9–1200 ng/L) and 93% of animal milk (median: 125.5, range: 10.7–4158 ng/L)
- 41% of infants exceeded provisional maximum tolerable daily intake limits (median: 0.67, 0.26–80.7 ng kg^–1^ bw day^–1^)
- 100% of mothers exceeded AFB1 PMTDI limits due to consumption of rice and flour (mean: 75.8, range: 35.2–318.2 ng kg^–1^ bw day^–1^) and 80% exceeded cut‐offs due to milk consumption
- Breast milk remains the optimal source of nutrition for infants although non‐exclusively breastfed children may be exposed due to contamination in the stable food supply

## 1 INTRODUCTION

Mycotoxins are secondary metabolites of fungi and are found in 25% of the global food system, resulting in annual losses of over 1 billion metric tons of food and feed products (Smith et al., 2016; Wu et al., 2014). In addition to being known carcinogens and genotoxins, literature suggests that mycotoxins may also be associated with infant growth faltering and stunting (Tesfamariam et al., 2019). Mycotoxins are found in a variety of food commodities such as maize, wheat, rice and animal source products, in addition to breast milk (Bayman & Baker, 2006; Deepa & Sreenivasa, 2017; Shirima et al., 2013; Smith et al., 2016; Sobrova et al., 2010; Wild & Gong, 2010). Thus, breast milk is a potential source of dietary exposure for infants and young children (Coppa et al., 2019; Fakhri et al., 2019). There are hundreds of different mycotoxins, but aflatoxins (AFs), ochratoxins (OTs), deoxynivalenol (DON) and fumonisins (FUM) are of particular consequence to public health (Warth et al., 2016).

Aflatoxins are produced by *Aspergillus flavus* and *Aspergillus parasiticus* fungi, and AFB_1_ is the most prevalent and toxic metabolite, classified as a group 1 carcinogen, known to cause cancer in humans (IARC [International Agency for Research on Cancer], 1993, 2012). Ochratoxins are produced by *Aspergillus ochraceus* and *Aspergillus penicillum* (Bayman & Baker, 2006; Smith et al., 2016). OTA is classified as a group 2B agent, possibly carcinogenic to humans (IARC, 1993). Deoxynivalenol is produced by *Fusarium graminearum* and *Fusarium culmorum* and is classified as a group 3 agent, with inadequate evidence for human carcinogenicity (IARC, 1993). Fumonisins are another important group of mycotoxins produced by *Fusarium verticillioides* (*Fusarium moniliforme*) and found predominantly as FB_1_ (70%) in commodities (Deepa & Sreenivasa, 2017). Fumonisins are classified as group 2B carcinogens (IARC, 1993, 2002).

Breast milk contains elements essential for child health, growth and development (World Health Organization, 2019). During early infancy, the primary exposure source to mycotoxins is breast milk, where lactational transfer of mycotoxins occurs via maternal diet (Warth et al., 2016). AFM_1_, also known as milk aflatoxin, is a hepatic hydroxylation by‐product of AFB_1_. It is tenfold less toxic and, although previously classified as a group 2B carcinogen, has in more recent IARC monographs been re‐evaluated to a group 1 carcinogen (IARC, 1993, 2002, 2012). AFM_1_ and OTA have been detected in breast milk samples from across the globe (Coppa et al., 2019; Fakhri et al., 2019). Infants and young children may experience the adverse effects of mycotoxin exposure up to three times greater than adults because of their larger intake/body weight ratio, higher metabolic rate and lower capacity to detoxify these contaminants (Assuncao et al., 2015; Hulin et al., 2014; Sherif et al., 2009).

Few studies have linked mycotoxin concentrations in breast milk with maternal diet to understand the role of the food system as a source of exposure (Galvano et al., 2008; Ortiz et al., 2018). To the best of our knowledge, no studies from India and only one from the South Asia region have reported on mycotoxin concentrations in breast milk (Khan et al., 2018).

Therefore, the aim of this cross‐sectional observational study was to quantify co‐occurring mycotoxins in an assortment of commonly consumed cereal crops (AFB_1_, FB_1_ and DON), commercial infant formula (AFB_1_) and 100 breast milk samples (AFs B_1_, B_2_, G_1_, G_2_, M_1_, M_2_, and OTs A, B) from rural and peri‐urban communities in Haryana, India. These data were used to understand determinants of breast milk mycotoxin exposure, characterize risk among mothers and children and explore variations in levels of mycotoxins across seasons (over the period of a year) and locations (peri‐urban vs. rural).

## 2 METHODS

### 2.1 Study subjects

The work presented here involved secondary analysis of data collected as part of a larger NIH R21 study on maternal malnutrition and lactation performance. The parent study was conducted in peri‐urban and rural areas within Faridabad district of the north Indian state of Haryana, with the aim to examine the role of maternal nutritional status on lactation performance and breast milk composition. The total population of Faridabad district is 1,809,733 (2011 census), and it comprises 20 primary health centres (PHCs), with a catchment population of 30,000–40,000 per PHC. According to the National Family Health Survey – 4 (2015‐2016), approximately 34% of children in this district are stunted, 21% are wasted and 16% of women have body mass indices of <18.5 kg/m^2^.

Participants for the study were recruited from the catchments of four PHCs. Mothers with infants 2–4 months of age were enlisted into the parent study from an existing pregnancy surveillance database, and all lactating mothers between the ages of 18–45 were eligible for enrolment. Exclusion criteria included not breastfeeding, consumption of any form of tobacco and those who were not likely to stay in the region for a period of 2 weeks post enrolment. Written informed consent was obtained from participants. The study protocol extended over a period of 14 days and contact with the household was established to obtain information about socio‐economic status, demographic details, food insecurity and infant morbidity. A 24‐h dietary recall questionnaire was also administered.

### 2.2 Breast milk sample collection

Breast milk samples were collected at the participant's household on Day 1 post enrolment. A cross‐sectional sample of participants was enrolled over the course of 12 months (July 2017 to June 2018) to capture seasonal variations. Milk samples were collected in a sterile acid washed polypropylene specimen container (Genaxy). Respondents were trained on how to manually hand‐express and provide a full sample of at least 30 ml of milk from one breast. Breast milk was gently inverted, aliquoted and stored at 2–8°C. Samples were transported to the local laboratory in cooler boxes and maintained at −80°C. Stored samples were shipped to the Central Food Technological Research Institute (CSIR‐CFTRI), Food Safety and Analytical Quality Control (FSAQCL) laboratories in Mysuru, India, for analysis of mycotoxins. Samples were shipped on dried ice with temperature monitoring and maintained at −80°C prior to analysis. All breast milk samples were thawed prior to processing, and each sample underwent only one freeze thaw cycle.

### 2.3 Food sample collection

We randomly selected 10 villages stratified to represent rural and peri‐urban communities from where breast milk, animal milk and food samples were collected. Additionally, food items were sampled from seven local retail markets and three wholesale markets accessed by participants in study communities. Samples were collected at three time points, namely, in May (summer), August (monsoon) and November (fall) 2018, to examine seasonal effects. We sampled from the same shops during each round of data collection, where feasible and longitudinally sampled from the same communities and markets at all three data collection time points.

Major crops produced in the north Indian state of Haryana include wheat, barley (rabi, spring harvest), rice, sorghum, pearl millet, maize and pulses (kharif, fall harvest) (Agri Haryana, 2018). In total, we collected 100 g of rice, wheat, flour, chilies, maize, pearl millet, sorghum, groundnuts, and barley samples (*n* = 298) and 30 commercial packaged and locally produced buffalo milk samples. Food samples were collected by asking shopkeepers to provide 100‐g samples of items, as they would to other customers. Ten commercial infant formula samples were collected in May 2019, to examine AFB_1_ levels. Details of sample sizes for food items collected by season are presented in Tables S1a and S1b.

### 2.4 Human and animal milk aflatoxin and ochratoxin analysis

#### 2.4.1 Chemicals and reagents

Standards for AFB_1_, AFB_2_, AFG_1_ and AFG_2_ were purchased from Sigma‐Aldrich (Bangalore, India) and standards (STDs) for AFM_1_, AFM_2_, OTA, OTB, internal standards, AFB_1_‐D_3_ and OTA‐D_5_ were purchased from Toronto Research Chemicals (TRC), Toronto, Canada. Purity of all standards and deuterated standards was ≥98%. High purity MS grade solvents (water and acetonitrile [ACN]) were procured from Honeywell, Bangalore, India. Centrifugal membrane filters (0.45 μm, PVDF) were obtained from Thermo‐Fisher Scientific (Bangalore, India). Formic acid and ammonium acetate were obtained from Sigma‐Aldrich (Bangalore, India). Standards were individually weighed and distributed into 1 mg/ml stocks in ACN and further diluted to 10 μg/ml aliquots in ACN and stored at −80°C. Working stock solutions ranged in concentration from 1 μg/ml for AFB_1_, AFB_2_, AFG_1_, OTA, to 0.5 μg/ml for AFM_1,_ AFM_2_ and OTB and 10 μg/ml for AFG_2_.

#### 2.4.2 Sample preparation

Aliquoted breast milk samples (1 ml) were allowed to thaw on top of ice and transferred to 15 ml centrifuge tubes. Ice‐cold ACN (2 ml) with 2% formic acid was then added, and 10 μl of internal standards were spiked on top. The sample was vortexed for 1 min and kept on ice for 5 min to allow for complete protein precipitation. Next, 300 μl of concentrated ammonium acetate (10 g/ml) solution was added. The sample was sonicated in a water bath sonicator for 10 min and kept on ice for 5 min and then centrifuged (5 min, 3800×*g*) to separate the ACN layer.

Approximately 1.5 ml of ACN from the supernatant was transferred to 2‐ml Eppendorf tube and dried in a speed vacuum. The final reconstitution was done with 100 μl of 50% ACN and filtered through a 0.45‐μm PVDF membrane centrifugal filter (5 min, 1500×*g*). The top 80 μl from each sample was transferred to HPLC vials and 10 μl from each was injected for analysis of mycotoxins in milk using the UHPLC‐MS/SRM method. A seven‐point calibration curve (rangesof 15.6–1000 ng/L for AFB_1_, AFB_2_, AFG_1_ and OTA; 7.8–500 ng/L for AFM_1_, AFM_2_ and OTB and 78–5000 ng/L for AFG_2_) was prepared on a daily basis, in addition to quality controls at limit of quantification (LOQ), low, medium and high‐quality control levels. All animal milk samples were analysed using UHPLC‐MS/MS in a manner similar to the breast milk samples, described above.

#### 2.4.3 Instrumental analysis

Sample analysis was conducted using a Sciex QTRAP 6500 (Sciex Singapore) mass spectrometer, with a turbo V ion source. The mass spectrometer was coupled to an Agilent 1290 infinity II UHPLC system (Agilent Technologies India Pvt. Ltd., India) and equipped with a column oven (set to 40°C), an auto‐sampler and thermo‐controller (set to 10°C). Mobile phase solvent A was water (10‐mM ammonium acetate, 0.1% formic acid) and solvent B was ACN (0.1% formic acid). A C‐18 column (2.1 × 100 mm, 1.8 μm, Agilent, Inc.) was used for separation of mycotoxins. We used an optimized gradient to achieve maximum separation (0–3 min—10%B; 3–15 min—10%B to 80%B; 15.1–17 min—100%B; 17.1–22 min—10%B) at 200 μl/min flowrate. Injection volume (10 μl) was kept constant throughout these analyses. Spray voltage was set at 5500 V, curtain gas at 30 PSI, temperature at 500°C, gas 1: 30 PSI, gas 2: 50 PSI, EP: 10 V. A scan time of 50 ms per transition was used in positive ion mode. Detailed tandem mass spectrometry scans (MS/MS) were obtained using infusion of 10 μg/ml solution of each mycotoxin in ACN and conducted in the syringe pump at a flow rate of 10 μl/min. We monitored the precursor ion and collision‐induced dissociation for each metabolite, which was used to generate details about product ions. We scanned quadrupole 3 to obtain product ions from m/z 50–500 with a cycle time of 1 s. We further optimized DP, CXP and CE for each intense product ion (Table S2).

### 2.5 Food sample AFB_1_, FB_1_ and DON analysis

#### 2.5.1 Sample preparation

Food samples were analysed using direct and indirect competitive enzyme‐linked immunosorbent assays (ELISA). Approximately 100 g of each food sample was ground to a fine powder using a Kenstar Senator blender (Kenstar, Gurgaon, India). Next, 100 ml of 70% methanol (v/v‐70 ml absolute methanol in 30‐ml distilled water) containing 0.5% KCl was added to 20 g sample powder in an Erlenmeyer flask. For DON extraction, 100‐ml deionized H_2_O was used in place of methanol, in accordance with the kit manufacturer's protocol. Extracts were incubated at room temperature for 60 min on a revolving shaker (250 rpm), filtered through Whatman no. 4 filter paper into a fresh tube and stored at 4°C until ELISA analysis. A similar protocol was used to prepare a toxin‐free sample extract (healthy groundnut), which served as a realistic crop matrix used for dilution of AFB_1_ and FB_1_ standards and as a negative control.

#### 2.5.2 ELISA assay procedures

For AFB_1_ and FB_1_, we conducted indirect competitive ELISA according to the protocols developed by ICRISAT (Reddy et al., 2001). Between each step of the protocol, contents were decanted and the plate washed three times with a PBST wash buffer. ELISA plates were coated with 150‐μl AFB_1_‐bovine serum albumin (BSA; 100 ng/ml) for AFB_1_ ELISAs, or FB_1_‐BSA (500 ng/ml) for FB_1_ ELISAs, both prepared in carbonate buffer (100 ng/ml) and incubated at 37°C for 1 h. Following this, blocking was conducted by adding PBST to each well and incubating at 37°C for 30 min. Standards (AFB_1_: 25‐0.097 ng/ml; FB_1_: 6‐0.047 μg/ml) were prepared in 10% toxin‐free extract with 7% methanol, and 100 μl was added to each well of the plate. Next, 100 μl of diluted sample extract (1:10 in PBST‐BSA) and 50 μl of antiserum diluted in PBST‐BSA (1:6000 for AFB1; 1:5000 for FB_1_) were added to all wells and incubated at 37°C for 1 h. Enzyme conjugation was done by adding 150‐μl anti‐rabbit‐IgG‐ALP (1:4000 in PBST‐BSA) to all wells and incubated at 37°C for 1 h. Finally, *p*‐nitrophenyl phosphate substrate prepared in 10% diethanolamine was added, leading to colour development in 20 min. Absorbance was read at 405 nm using a Bio‐Rad iMark microplate reader (Bio‐Rad Laboratories, CA, USA). The assays were validated previously, with LOQ 1 μg/kg (93% recovery) and 10 μg/kg (recoveries between 61 and 84%) for AFB_1_ and FB_1_, respectively (Barna‐Vetro et al., 2000; Reddy et al., 2001). As these assays were already validated and method development was not among our aims, spike and recovery tests were not performed in this study.

For DON analysis, we used a commercially available test kit (HELICA Biosystems, CA, USA) to perform direct competitive ELISAs. The assay was conducted and performed in accordance with manufacturer's instructions. First, 200 μl of the conjugate solution was mixed with 100 μl sample extract or DON standard (10–0 μg/ml) in a 96‐well dilution plate. After dilution, 100 μl of the contents from each dilution well were transferred to the corresponding antibody coated microtiter well of the kit's test plate and incubated at room temperature for 15 min. The contents of the test plate were discarded, and the plate was washed three times with a PBST wash buffer. We then added 100 μl of the substrate reagent to each well and incubated at room temperature for 5 min. Finally, 11 μl of Stop Solution was added to the plate in the same sequence as the substrate reagent. Absorbance was read at 450 nm using the same instrument as described above. The LOQ for DON was 10 μg/kg (HELICA Biosystems, CA, USA).

#### 2.5.3 ELISA data analysis

All samples were assayed in duplicate on the ELISA plates. Optical densities (ODs) were recorded and processed using Microplate Manager 6 software (Bio‐Rad Laboratories, CA, USA). For AFB_1_ and FB_1_, second‐order polynomial standard curves were generated for each plate, plotting Log_10_ values of standard concentrations on the *y*‐axis and OD values on the *x*‐axis. For DON, standard curves were generated according to manufacturer's instructions by calculating % bound (%B/Bo) for each standard and plotting Logit %B/Bo on the *y*‐axis against Log_10_ DON concentration on the *x*‐axis. For all toxins, the standard curves were used to compute sample concentrations by interpolation, taking all sample dilution factors into account. Samples with OD values outside the OD range of the standards were serially diluted and re‐analysed until their OD values were within range.

### 2.6 Dietary intake data for mothers and infants

A food frequency questionnaire (FFQ) and multiple pass 24‐h dietary recall were collected from each respondent during a household visit. A semi‐quantitative FFQ validated as part of the Indian Migration Study (IMS) was implemented, and items were divided into groups, namely, *breads and rotis*; *grains and staples*; *dairy, eggs and animal fat*; *fruits and vegetables*; *lentils*; *meats and sweets and snacks* (Bowen et al., 2012). The 24‐h recall data were entered into DietCal, a software developed by the All India Institute of Medical Sciences (2015).

Infant breast milk intake was assessed using deuterium oxide dose‐to‐mother technique, where a single dose of 30 g of 99.8% deuterium oxide (D_2_O) was administered to lactating mothers on Day 0 of enrolment (International Atomic Energy Agency [IAEA], 2010). Saliva collection procedures were based on methods prescribed by the IAEA human health series (Number 7) (IAEA, 2010). Samples were stored in 2‐ml acid washed cryogenic vials at −20°C. Analysis of saliva samples was conducted using Fourier transform infrared spectrometry, which provided the exact volume of breast milk consumed by each child (L/day). Infant body weight was measured using a SECA 385 (SECA GmbH & Co., Hamburg, Germany; accuracy 20 g), and length was measured using a SECA 417 (SECA GmbH & Co., accuracy 1 mm). All measures were taken in duplicate.

### 2.7 Statistical analysis of data

All statistical analyses were conducted in SAS 9.4 (SAS Institute, Cary, NC). Maternal and demographic characteristics are expressed as percentages and means ± SD. A household wealth index was derived using principal components analysis and the Household Food Insecurity and Access Scale was used to categorize households by food security status (Food and Nutrition Technical Assistance, 2007).

Breast milk and food sample mycotoxin results are presented as medians and interquartile ranges (IQRs). Left censored data were handled by replacing the values for each mycotoxin, below the limit of detection with the medium bound value or ½ that of LOD (EFSA, 2010).

Non‐normally distributed data were log transformed. TOBIT (PROC QLIM) or censored regression models were generated to identify factors significantly associated with breast milk mycotoxin concentrations using a two‐step approach. Variables with a *p* < 0.1 in bivariate analyses were included in multivariable models by category of determinants (sociodemographic, dietary, environmental), controlling for maternal age and wealth status. A *p‐*value < 0.05 was considered statistically significant. For analysis of dietary determinants, we used frequencies for consumed versus not consumed. Analyses of variance and Kruskal–Wallis tests were used to assess differences in mean concentrations of AFB_1_ and FB_1_ in food items by season and location of collection (rural/village level markets, peri‐urban/retail markets and urban/wholesale markets).

### 2.8 Dietary exposure assessment and risk characterization

Estimated daily intake (EDI) of AFB_1_ and AFM_1_ for mothers via consumption of a variety of food items and animal milk and of AFM_1_ for infants through breast milk were calculated using a deterministic exposure assessment approach (Assuncao et al., 2015; Cantu‐Cornelio et al., 2016; Ortiz et al., 2018). EDIs were calculated using the equation below and reported as ng kg^−1^ bw day^−1^ for relevant mycotoxins in each food item for which consumption data were available (Ortiz et al., 2018).

$$\text{Estimated daily intake}=\text{average concentration of mycotoxin}\;\left(\mathrm{ng}/\mathrm{kg}\;\text{or}\;\mathrm{ng}/L\right)\times \text{consumption of item}\;\left(\mathrm{kg}/\mathrm{day}\;\text{or}\;L/\mathrm{day}\right)÷\text{body weight}\;\left(\mathrm{kg}\right).$$

Average concentrations of mycotoxins in food items and milk were derived from study samples. Average consumption of food items was derived from 24‐h recall data for mothers and deuterium oxide (D_2_O) results for infant breast milk volume consumed. Daily intake values were compared with reference dose values as part of risk characterization. As aflatoxins are carcinogenic compounds, exposure at any level is considered unsafe and levels of such substances should be as low as reasonably achievable (ALARA) (JECFA/WHO, 2007). A provisional maximum tolerable daily intake (PMTDI) value of 1 ng kg^−1^ bw day^−1^ for aflatoxins has been proposed for children and adults without hepatitis B (Kuiper‐Goodman, 1995, 1998). The extent to which exposure exceeds these values was used to assess risk (Cunha et al., 2018).

The European Food Safety Agency (EFSA) has also proposed the use of margin of exposure (MOE) for risk analysis of aflatoxins. MOE represents the ratio between BMDL_10_ (benchmark dose lower confidence limit for 10% extra risk of liver tumour formation in rats amounting to 170 ng kg^−1^ bw day^−1^) and EDI (EFSA, 2005). Here, an EDI is considered of concern to public health if the MOE is lower than 10,000. MOE values do not quantify risk but are used to indicate a level of concern. A lower MOE indicates a higher level of concern (EFSA, 2005).

### 2.9 Ethical considerations

Ethics approval for this study was provided by the Indian Council for Medical Research‐ HMSC, Society for Applied Studies Research Ethics Board and Emory University Internal Review Board.

## 3 RESULTS

### 3.1 Analytical validation

The occurrence of six aflatoxins (AFB_1_, AFB_2_, AFG_1_, AFG_2_, AFM_1_, AFM_2_) and two ochratoxins (OTA, OTB) were investigated in human and animal milk samples. Method performance for the assay was in accordance with guidelines set by the U.S. FDA (Guidelines for Bioanalytical Method Validation, 2018), and results are presented in Table S3. All analytes presented a good linear response (*R* ^2^ between 0.9991 and 0.9999), and recoveries were >75% for standards spiked on top of milk and >88% for standards spiked to water.

### 3.2 Population characteristics

Sociodemographic characteristics of households, in addition to details about mothers and children in our sample, are presented in Table 1.

**TABLE 1:** Household, maternal and child characteristics of *N* = 100 participants in Haryana, India

**Household characteristics (*N*)**	**%**
Religion	
Hindu	71
Muslim	27
Other	2
Caste	
Scheduled caste	26
Other backward caste (OBC)	50
Other caste	24
Paternal occupation	
Not working/retired/unemployed	5
Cultivator (own land)	2
Business/petty trader/self‐employed	20
Salaried employee	49
Other	24
Socio‐economic status	
Low	33
Middle	34
High	33
Residence	
Peri‐urban	77
Rural	23
Food insecurity	
Food secure	82
Food insecure	18
**Maternal characteristics**	**Mean (±SD)**
Maternal age (years)	24.9 (3.6)
Maternal education (years)	6.8 (5.1)
Parity	2.3 (1.3)
Anthropometry	
Weight, kg	52.0 (9.97)
Height, cm	152.7 (6.0)
BMI, kg/m^2^	22.0 (3.7)
BMI categories, kg/m^2^	
Underweight (<18.5)	11
Normal (18.5–25)	69
Overweight (25–30)	17
Obese (>30)	3
Current dietary patterns	
Vegetarian	36
Nonvegetarian	53
Eggetarian	11
**Child characteristics**	**Mean (±SD) (%)**
Child sex	
% male	51
% female	49
Child age (months)	3.07 (1.99, 4.05)
Child nutritional status	
% stunted (length‐for‐age *z* score < −2)	18
% underweight (weight‐for‐age *z* score < −2)	16
% wasted (weight‐for‐length *z* score < −2)	8
Current breastfeeding practices	
Currently exclusively breastfeeding	28

### 3.3 Occurrence of aflatoxins and ochratoxins in breast milk

AFM_1_ was detected in 41 breast milk samples. Other mycotoxins including AFB_2_, AFG_2_, AFM_2_, OTA and OTB were also detected at levels above the LOD in 43%, 19%, 18%, 13% and 42% of samples, respectively.

Contamination levels for AFM_1_ in milk ranged between 0 and 1200 ng/L. Only one sample was detected with AFM_1_ levels above Food Safety and Standards Authority of India (FSSAI) set limits in animal milk (500 ng/L) (Table 2). There are currently no cut‐offs for aflatoxins in breast milk samples as these are not commercially regulated.

**TABLE 2:** Concentrations (ng/L) of aflatoxins and ochratoxins in human breast milk (*n* = 100)

	LOD	LOQ		Median^b^	Interquartile range (IQR)				Maximum regulatory limits^d^
Mycotoxin	(ng/L)		% samples > LOD^a^	(ng/L)		Min^c^	Max	*N* > regulatory limits	(ng/L)
Aflatoxin B_1_	15.6	31.3	8	22.9	7.4	7.8	27.1		100
Aflatoxin B_2_	15.6	31.3	43	43.0	49.0	7.8	265.0		‐
Aflatoxin G_1_	15.6	31.3	5	23.7	7.3	7.8	45.3		‐
Aflatoxin G_2_	156	313	19	121.0	71.7	39.0	241.0		‐
Aflatoxin M_1_	7.8	15.6	41	13.7	16.3	3.9	1200.0	1	25 500^e^
Aflatoxin M_2_	7.8	15.6	18	15.0	9.1	3.9	33.9		‐
Ochratoxin A	15.6	31.3	13	20.0	13.2	7.8	39.8	‐	500
Ochratoxin B	7.8	15.6	42	11.9	23.8	3.9	62.2		‐

^a^ Number of samples below the limit of detection and assigned a value of 0.5 LOD.

^b^ Median of values > LOD.

^c^ Values corresponding to 0.5 LOD for each mycotoxin (Ortiz et al., 2018).

^d^ Maximum regulatory limits set for infant formula, follow‐on formula, baby foods for infants and young children by the European Union.

^e^ Maximum regulatory limits set for animal milk.

Details of individual food items composing each group in our FFQs and median frequencies of daily and weekly intake are presented in Table S4. TOBIT regression analyses of the determinants of AFM_1_ exposure in breast milk samples showed maternal consumption of items such as tandoori roti and stuffed parathas, both wheat flour based Indian flatbreads to be significantly (*p* < 0.05) associated with an increase in breast milk concentrations of AFM_1_. This trend remained significant for both bread items after adjusting for maternal age and household wealth index (Table 3).

**TABLE 3:** Determinants of AFM_1_ (including outlier) exposure in breast milk samples

	Univariate TOBIT regression			
	**AFM**_**1**_ **β (95% CI)** ^b^	***p* value**		
**Maternal age**	0.93 (0.15, 7.56)	0.26		
**Wealth index**				
Low	0.78 (0.25, 12.45)	0.65		
Middle	0.87 (0.25, 12.39)	0.80		
High	*REF*			
	**Univariate TOBIT regression**		**Multivariable TOBIT regression**	
	**AFM**_**1**_ **β (95% CI)** ^b^	***p* value**	**AFM**_**1**_ **β (95% CI)** ^b^	***p* value** ^c^
**Dietary variables**				
Tandoori roti	3.86 (0.27, 13.43)	**0.03**	4.10 (0.27, 13.45)	**0.027**
Stuffed Paratha	3.93 (0.25, 12.43)	**0.015**	3.93 (0.25, 12.35)	**0.014**
Rice	0.45 (0.22, 11.18)	0.08	0.45 (0.22, 11.19)	0.08
Apple	2.65 (0.23, 11.72)	0.05	2.42 (0.24, 11.91)	0.09

*Note*: Bold numbers show the statistically significant coefficients.

^a^ Multivariable models adjusted for maternal age and wealth index.

^b^ Log transformed values back‐transformed (presented in table) for interpretation of β coefficient.

^c^ No significant associations with sociodemographic and environmental variables.

No statistically significant trends were observed for sociodemographic variables (maternal age, parity, years of schooling), maternal biological characteristics (weight, body fat, body mass index, fat mass index), household characteristics (religion, caste, HH wealth index, food security index, region of residence [peri‐urban/rural], paternal occupation) and/or environmental variables (location of procurement of staple crops, self‐reported instances of insect infestation and moisture in household storage of crops).

**FIGURE 1:**
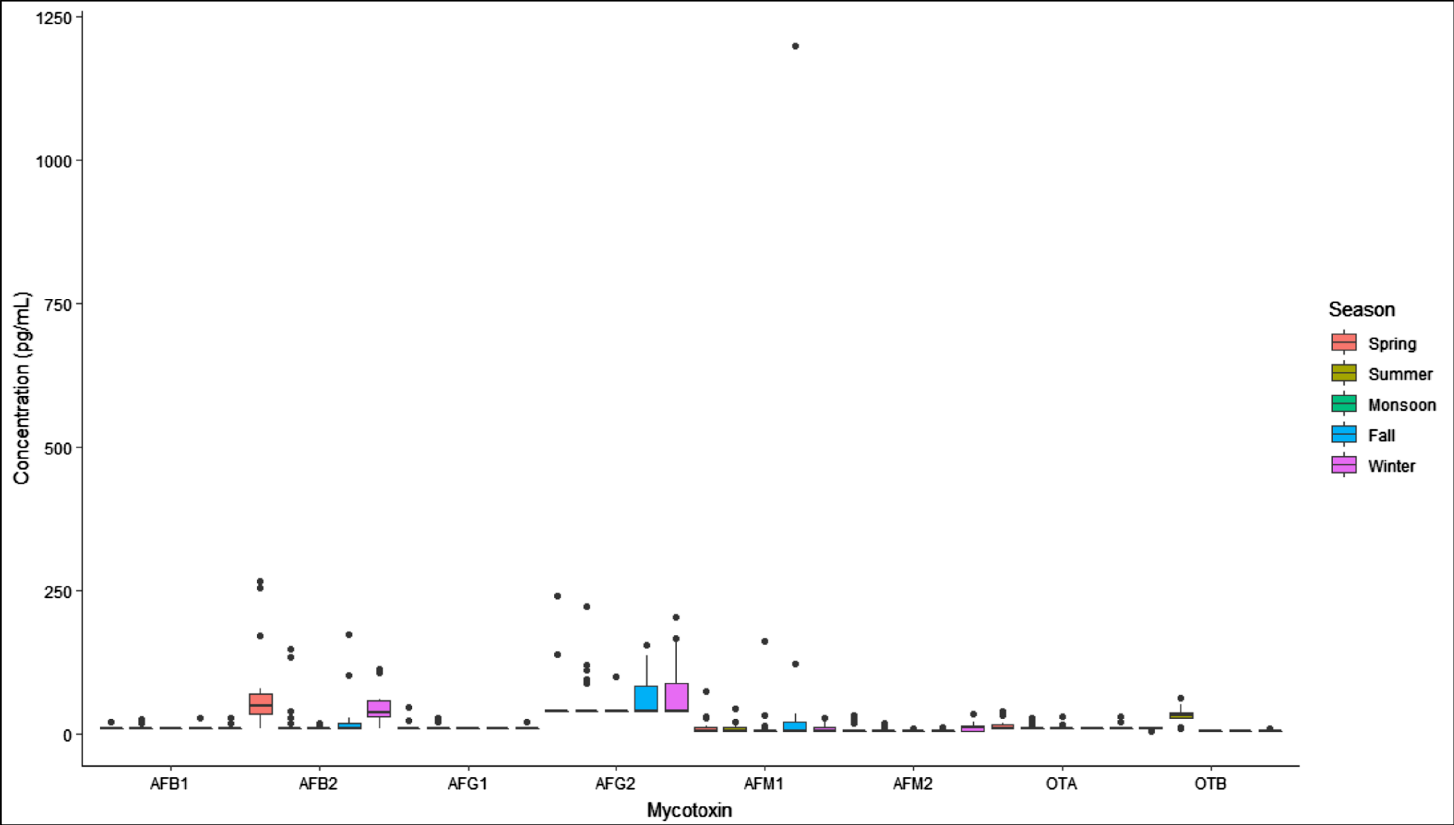
Breast milk Aflatoxin M_1_ concentrations by month^a^. ^a^N per month, Monsoon: ‐ July: 7, Aug: 8, Sept: 9, Fall: ‐ Oct: 8, Nov: 9, Winter: ‐ Dec: 9, Jan: 8, Spring: ‐ Feb: 9, March: 8, Summer: ‐ April: 8, May: 9, June: 8

**FIGURE 2:**
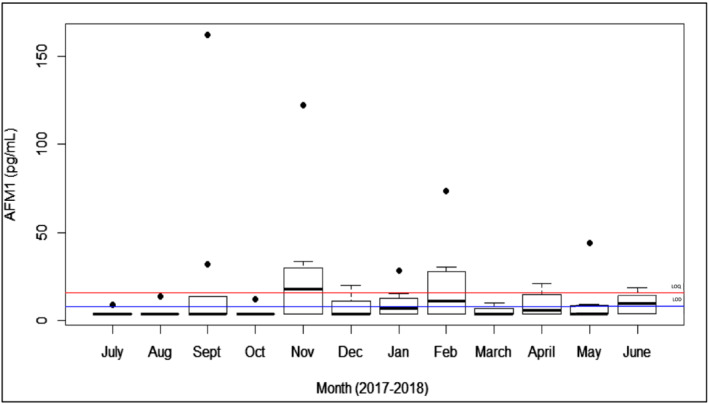
Breast milk mycotoxin concentrations by season^a,b^. ^a^N per month, Monsoon: ‐ July: 7, Aug: 8, Sept: 9, Fall: ‐ Oct: 8, Nov: 9, Winter: ‐ Dec: 9, Jan: 8, Spring: ‐ Feb: 9, March: 8, Summer: ‐ April: 8, May: 9, June: 8. ^b^N for AFM_1_: 99, after removing outlier ([AFM_1_] of 1200 pg/ml

Concentrations of all mycotoxins examined in breast milk by season are shown in Figure 1. We did not see statistically significant seasonal differences in values of AFM_1_ in breast milk samples across the 12‐month span for which data were collected (Figure 2). Mean concentration of OTA in our study was 23.15 (±7.78) ng/L, and only two samples were above the limit of quantification. Other mycotoxins were detected at levels less than the LOQ in our study and have therefore not been considered for further statistical analyses. Median (IQR) concentrations of mycotoxins in breast milk are presented in Table 2.

#### 3.3.1 Occurrence of aflatoxins and ochratoxins in animal milk

AFM_1_ was detected across all 30 samples of animal milk (Figure S2) at levels above the 7.8 ng/L, limit of detection. Median (IQR) values by season were as follows: summer—102.45 (178.80); monsoon—50.35 (165.70); fall—389.21 (1646.33). Eight (27%) animal milk samples were over the 500 ng/L regulatory limit set by the FSSAI. Other mycotoxins including AFB_1_ (*n* = 19), AFB_2_ (*n* = 23), AFG_1_ (n = 1) and AFM_2_ (n = 23) were seen in several samples at levels above the limit of detection (Figure S2). OTA and OTB were not detected in any of the animal milk samples in this study.

### 3.4 Occurrence of Aflatoxin B_1_, Fumonisin B_1_ and DON in food samples

Aflatoxin B_1_ was detected across a range of food items at all three seasonal time points, and several food samples were above FSSAI set regulatory limits. Median sample AFB_1_ concentrations by food items are presented in Table S3. We saw statistically significant (*p* < 0.05) variations in AFB_1_ levels across seasons for wheat, sorghum, flour, groundnuts, rice and pearl millet, with highest mean concentrations observed during the monsoon collection period (Tables S1a and S1b). Negligible levels of AFB_1_ were detected in 10 commercial infant formula samples collected in our study.

Fumonisin B_1_ was analysed only in maize, pearl millet and sorghum, given the known vulnerability of these commodities to infestation by implicated fungi. The toxin was detected in these commodities across the three seasonal time points (Table S1). Samples were above regulatory limits set by the European Union for maize (0.2 mg/kg) at all three time points. We saw statistically significant seasonal trends for mean concentrations of FB_1_ in pearl millet and sorghum samples. Both pearl millet and sorghum are harvested in the fall, which likely explains non‐detected levels of FB_1_ contamination during the fall collection period. We did not see any statistically significant trends for variation in mean concentrations of AFB_1_ or FB_1_ among rural and peri‐urban sites and between types of markets, namely, village, mid‐retail and wholesale levels. Deoxynivalenol was not detected in any of the food samples in our study, potentially owing to low disease pressure by *F*. *graminearum* and the generally warm climate in the study region.

### 3.5 Deterministic exposure assessment and risk assessment

Maternal exposure assessment to mycotoxins through intake of animal milk, flour and rice was conducted. Data from the 24‐h recall show that 93% of women in our sample consumed milk at an average volume of 0.33 L/day (±0.30), 92% consumed wheat flour at 0.24 kg/day (±0.10) and rice was consumed by 21% of our sample at 0.19 kg/day (±0.14). Mean maternal estimated daily intake of AFM_1_ due to milk was 3.58 (range: 0.42–17.74) ng kg^−1^ bw day^−1^. Mean estimated daily intake for AFB_1_ via flour was 82.3 ng kg^−1^ bw day^−1^ and 46.4 ng kg^−1^ bw day^−1^ via rice (Table 4).

**TABLE 4:** Estimated daily intake (EDI) using a deterministic approach for mothers and infants

Commodity		Mean intake	SD	P50	P75	P90	P95	P97.5	P99
		(kg/day)							
Flour	Mother	0.24	0.10	0.24	0.30	0.36	0.40	0.47	0.49
Rice		0.19	0.14	0.15	0.20	0.40	0.50	0.50	0.50
Animal milk		0.33	0.30	0.25	0.45	0.66	0.98	1.02	1.18
Breast milk	Infant	0.75	0.17	0.73	0.86	0.96	1.01	1.12	1.25
**Commodity**		**Mean estimated daily intake AFB**_**1**_ **/AFM**_**1**_	**SD**	**P50**	**P75**	**P90**	**P95**	**P97.5**	**P99**
		**(ng/kg bw/day)**							
Flour	Mother	82.3	36.9	80.6	106.7	131.3	149.2	162.5	172.0
Rice		46.4	35.8	36.7	67.1	98.2	121.9	125.1	127.0
Animal milk		3.58	3.20	2.58	5.09	7.40	9.62	11.55	13.0
Breast milk	Infant	3.04	14.4	0.67	1.70	3.29	4.99	15.15	80.7

Using a deterministic risk assessment approach, 80% of women exceeded this cut‐off for AFM_1_ due to milk consumption; 100% of our sample significantly exceeded the PMTDI level of 1 ng kg^−1^ bw day^−1^ for AFB_1_ due to consumption of rice and wheat flour (Kuiper‐Goodman, 1998; Yogendrarajah et al., 2014). MOE values for maternal aflatoxin exposure from flour (range: 0.90–17.21), rice (range: 1.33–17.54) and milk (range: 9.58–404.53) were also lower than 10,000 indicating that exposure to AFB_1_ and AFM_1_ in the food system are of priority for risk management actions (Ortiz et al., 2018).

Mean estimated daily intake value of AFM_1_ from breast milk for infants in our study was 3.04 ng kg^−1^ bw day^−1^, 41% of the sample exceeded PMTDI levels with upper percentiles of milk consumption exceeding thresholds by 3–80 folds (P90–P99). Approximately 16% of infants in our sample consumed animal milk, 7% consumed formula and 5% received food grain and/or porridge made of grain, in addition to breast milk. Thus, exposure to mycotoxins in dairy milk and via contaminated complementary foods may be of concern among children in this population.

## 4 DISCUSSION

We found AFM_1_ in 41 out of 100 breast milk samples analysed for mycotoxins in India at concentrations ranging between 7.98 and 1200 ng/L. Studies from countries including Iran, Brazil, Turkey, Italy, Egypt, Jordan, Germany and Nigeria have quantified AFM_1_ in breast milk samples at concentrations ranging between 0 and 19,000 ng/L (Coppa et al., 2019). Fakhri et al. (2019) in a recent systematic review, noted an overall pooled concentration of AFM_1_ across 196 global studies to be 27.67 (95% CI: 26.67–28.67 ng/L) (Fakhri et al., 2019). Samples from our study show concentrations to be within the ranges described by others, although no other studies on breast milk mycotoxins from India currently exist in the literature. The overall prevalence of AFM_1_ in breast milk from studies conducted globally is highly variable and associated with mycotoxin contamination in food items and in turn maternal dietary patterns, which differ across countries (Abdulrazzaq et al., 2003; Cherkani‐Hassani et al., 2016; Coulter et al., 1984; Memis & Yalcin, 2019).

Our findings suggest that the consumption of breads and rotis among women in this population is associated with increased concentrations of AFM_1_ in milk. This is likely due to the presence of AFB_1_ in flour used to produce these Indian flatbreads and rotis. As an important caveat, we acknowledge that associations noted here may be spurious and larger sample sizes are needed to validate our findings.

Carry‐over of AFB_1_ from diet and excretion as AFM_1_ in breast milk is estimated to be between 0.1% and 0.4% (Zarba et al., 1992). Prior studies have found maternal consumption of cereals, peanut butter, vegetable oil, rice (Elzupir et al., 2012), cow milk (Mahdavi et al., 2010) and sausage (Jafarian‐Dehkondi & Pourradi, 2013) to be associated with increased concentrations of AFM_1_ in breast milk from women in Iran. A study conducted in Italy found that lactating women with high AFM_1_ (140 ng/L) in breast milk had consumed a large amount of corn meal‐based foods in substitution for cereal‐based food such as rice, pasta, bakery products and breakfast cereals (Galvano et al., 2008). Consumption of corn oil, peanuts, raw milk, beans and wheat meal have been associated with higher breast milk AFM_1_ in Egypt and Nigeria (Adejumo et al., 2013; El‐Tras et al., 2011; Polychronaki et al., 2006). Higher consumption of rice and chocolate have also been associated with AFM_1_ in milk (Bogalho et al., 2018).

We did not see any significant seasonal trends in concentrations of AFM_1_, likely due to a small per season sample size. Future research should examine maternal dietary patterns to understand the role of seasonal variation in sources of AFM_1_ exposure. Previous studies have shown associations between AFM_1_ concentrations in breast milk and season of sample collection, lower educational levels and stage of lactation (Adejumo et al., 2013; Bogalho et al., 2018; Polychronaki et al., 2006). Bogalho et al. (2018) noted that breast milk collected at early stages of lactation featured mean concentrations of AFM_1_ higher than samples collected 6–12 months post partum. Several additional determinants of lactational transfer of mycotoxins have been identified, including but not limited to maternal dietary diversity and hydration, frequency of infant feeding and breast infection (Warth et al., 2016).

There remains a dearth of knowledge about the lactational transfer rates of mycotoxins from blood to breast milk. Milk to plasma ratios for AFM_1_ were estimated to be 0.21 among nursing Egyptian mothers (Hassan et al., 2006), although this metabolite is transient in plasma, and concentrations of AFM_1_ in breast milk are higher at earlier stages of lactation (Degen et al., 2013; Polychronaki et al., 2006). We did not detect OTA in most breast milk samples in our study. Only a few studies from countries including Brazil, Iran, Chile, Italy and Turkey have documented OTA in breast milk at ranges between 0 and 13,111 ng/L (Coppa et al., 2019).

Our findings for AFM_1_ in animal milk samples are in concordance with a large nationally representative survey conducted by the FSSAI in 2018, which found 5.7% (*n* > 6,000) of milk samples in India to be contaminated with AFM_1_ (FSSAI, 2019). Other studies conducted in India have shown AFM_1_ contamination at ranges of 65–1,012 ng/L in milk products, 28–164 ng/L in liquid milk; and in animal milk samples from southern India at ranges between 100 and 3800 μg/L (Rastogi et al., 2004; Siddappa et al., 2012).

Our findings for AFB_1_ in food items are in alignment with others that have documented levels of aflatoxins in maize, groundnuts, spices, peanuts, corn, rice, soybeans, sorghum, cereals, and chilies at levels ranging between 0 and 46,000 μg/kg (Reddy et al., 2010). Higher levels of AFB_1_ in food samples collected during the monsoon season in our study are likely due to increased moisture levels and humidity, known to impact the growth of mycotoxigenic mould (Ojuri et al., 2019). A large multicentre study conducted in India found 40.3% of wheat samples collected from across the country to have AFB_1_ levels ≥5 μg/kg, with 16% of samples over the Indian permissible regulatory limit of 30 μg/kg, when that study was published (current legal limit in India is 15 μg/kg) (Toteja et al., 2006). Additional studies of items intended for animal feed from India have found aflatoxin contamination in groundnut cake, maize, millets, rice bran, sorghum, soybeans, sunflower and mixed feeds in excess of 10 μg/kg (Thirumala‐Devi et al., 2002).

Calculated daily intake values in children for AFM_1_ via breast milk in our study ranged between 0.26 and 80.7 ng kg^−1^ bw day^−1^, whereas others conducted globally have documented ranges between 0.003 and 917 ng kg^−1^ bw day^−1^, accounting for variations in prevalence and concentrations of mycotoxins across regions (Coppa et al., 2019). Cumulative estimated daily intake of AFB_1_ due to consumption of rice and flour, among mothers, ranged between 35.2 and 318.2 ng kg^−1^ bw day^−1^. Dietary Aflatoxin B_1_ exposure between 20 and 120 μg kg^−1^ bw day^−1^ for periods of 1–3 weeks or consumption of staple foods containing concentrations of AFs of 1 mg/kg or higher are suspected to cause acute aflatoxicosis and possibly death (JECFA, 2016). Overall levels of AFB_1_ exposure in our study are significantly below these thresholds.

Our findings for Fumonisin B_1_ in maize, sorghum and millet are in accordance with other studies that have documented FB_1_ and AFB_1_ in these items, in addition to poultry feed from India (Shetty & Bhat, 1997). We did not detect DON in our study; however, other studies conducted in northern India have detected the mycotoxin in upward of 30% of cereal crop samples collected, with 7% exceeding FSSAI set limits of 1 mg/kg (ranges: 0.01–4.73 mg/kg) (Mishra et al., 2013). More recently, a study conducted in India found 51.7% of food samples commonly consumed by infants and young children to be contaminated with DON at levels significantly above the JECFA prescribed PMTDI level of 1 μg/kg bw (Gummadiadala et al., 2016; JECFA, 2010).

Additional sources of dietary exposure to AFs and other mycotoxins not examined in this study are likely in this population and require further investigation, in conjunction with the additive and synergistic effects of such exposures on health (Smith et al., 2016). Overall maternal EDIs for AFB_1_ and AFM_1_ are higher than those in infants. This suggests that the toxic concentrations of mycotoxins are reduced as they move down the food chain. Breast milk, although a potential source of toxin exposure, is significantly less harmful to infants as compared with other dietary sources that may be contaminated at higher concentrations.

Although limited by a small sample size, we have used gold standard methods for the detection and quantification of mycotoxins in breast milk samples. We were also able to assess seasonality in relation to concentrations of mycotoxins at various levels of the food ecosystem and from breast milk within study communities.

We were not able to calculate estimated daily intake values for all foods consumed by mothers in our sample. It is therefore likely that cumulative exposure is higher than noted here, from other sources, in addition to exposure to other mycotoxins not quantified in our study. It is also critical to investigate mycotoxin exposure among infants who are not being exclusively breastfed, and those consuming weaning foods, as exposures are likely to be higher in these groups. Studies in East Africa have shown exposure to AFs and FBs in infant foods to be of concern among non‐exclusively breastfed children under 6 months of age (Magoha et al., 2016). Another limitation of this study is that the matrix effects of different commodities in the validated AFB_1_ and FB_1_ ELISA protocols have not been thoroughly explored. While it is unlikely that accounting for commodity matrix effects in food samples would qualitatively change our results or conclusions, evaluating and accounting for such effects may yield more precise estimates in future work.

Finally, there is a need for longitudinal cohort studies, complemented with total diets data to examine exposure to mycotoxins across the different stages of lactation, particularly in light of mixed evidence regarding their effects on birth outcomes including small‐for‐gestational age (SGA), infant growth faltering and stunting (Andrews‐Trevino et al., 2019; Hoffman et al., 2018; LeRoy et al., 2018; Passarelli et al., 2019).

## 5 CONCLUSION

Breast milk remains the best source of essential nutrients and immune factors for infants, and exclusive breastfeeding is the recommended standard of practice for children under 6 months (World Health Organization, 2019). Although AFM_1_ was detected in 41% of breast milk samples, concentrations of the aflatoxin remain low and below FSSAI set regulatory limits. Exposure assessments suggest levels of aflatoxins in food are higher than permissible limits. Women who consume flour‐based products such as bread are at increased risk of mycotoxin exposure, in this population. Between 80% and 100% of women in our sample had dietary intakes of AFs above recommended daily limits of exposure. Given a growing body of evidence showing dose‐dependent associations between the consumption of aflatoxins and liver cancer, other mycotoxins and cancers of the breast and cervix, more in‐depth evaluations of mycotoxin exposure and contamination across the food system, from feed to human, are prudent and timely (Claeys et al., 2020). Furthermore, human epidemiological studies remain the need of the hour to support evidence‐based public health strategies to mitigate mycotoxin contamination and their harmful effects on all humans, with an emphasis on vulnerable populations including mothers and children.

## CONFLICTS OF INTEREST

The authors declare that they have no conflicts of interest.

## CONTRIBUTIONS

RVM and MFY designed the study. RVM and KR developed the LC‐MS/MS method for analysis of mycotoxins in breastmilk. RVM conducted all breastmilk sample analysis. AJW assisted with design of food sample collection strategy and conducted all food sample mycotoxin analyses. ST and SR collected all breastmilk samples; RVM collected all food samples. RVM conducted all statistical analyses and drafted the manuscript. AJW, AWG, UR, ST, PBR, RM and MFY provided guidance and consultation during the study and critically reviewed this manuscript. RVM, MFY and RM procured funding for the study. All authors have read and approved the final version of this manuscript.

## Supporting information


**Figures S1** **a‐d):** Chromatograms for AFs & OTs in standards & human breastmilk
**Figure S2):** Mycotoxin concentrations in animal milk samples (*N* = 30)^a,b,c^
**Table S1a:** Concentrations of AFB_1_ in food items by season
**Table S1b:** Concentrations of FB_1_ in food items by season
**Table S2:** Selected Reaction Monitoring Table
**Table S3:** Performance characteristics of LC–MS/MS analytical method
**Table S4:** Median intake from FFQs of commonly consumed food items
